# Effective H_2_ Separation through Electroless Pore-Plated Pd Membranes Containing Graphite Lead Barriers

**DOI:** 10.3390/membranes10120410

**Published:** 2020-12-10

**Authors:** David Martinez-Diaz, Raúl Sanz, Alicia Carrero, José Antonio Calles, David Alique

**Affiliations:** 1 Department of Chemical, Energy and Mechanical Technology, Rey Juan Carlos University, C/ Tulipán s/n, 28933 Móstoles, Spain; david.martinez.diaz@urjc.es (D.M.-D.); alicia.carrero@urjc.es (A.C.); joseantonio.calles@urjc.es (J.A.C.); 2 Department of Chemical and Environmental Technology, Rey Juan Carlos University, C/ Tulipán s/n, 28933 Móstoles, Spain; raul.sanz@urjc.es

**Keywords:** hydrogen, composite membrane, palladium, electroless plating, intermediate layer, graphite

## Abstract

Hydrogen promotion as a clean energy vector could provide an efficient strategy for realizing real decarbonization of the current energy system. Purification steps are usually required in most H_2_-production processes, providing the use of Pd-based membranes, particularly those supported on porous stainless steel (PSS), important advantages against other alternatives. In this work, new composite membranes were prepared by modifying PSS supports with graphite, as an intermediate layer, before incorporating a palladium film by electroless pore-plating. Fully dense Pd layers were reached, with an estimated thickness of around 17 μm. Permeation measurements were carried out in two different modes: H_2_ permeation from the inner to the outer side of the membrane (in–out) and in the opposite way (out–in). H_2_ permeances between 3.24 × 10^−4^ and 4.33 × 10^−4^ mol m^−2^ s^−1^ Pa^−0.5^ with α_H2/N2_ ≥ 10,000 were reached at 350–450 °C when permeating from the outer to the inner surface. Despite a general linear trend between permeating H_2_ fluxes and pressures, the predicted intercept in (0,0) by the Sieverts’ law was missed due to the partial Pd infiltration inside the pores. H_2_-permeances progressively decreased up to around 33% for binary H_2_–N_2_ mixtures containing 40 vol% N_2_ due to concentration–polarization phenomena. Finally, the good performance of these membranes was maintained after reversing the direction of the permeate flux. This fact practically demonstrates an adequate mechanical resistance despite generating tensile stress on the Pd layer during operation, which is not accomplished in other Pd membranes.

## 1. Introduction

Human activities and lifestyles with a high industrialization level, excessive consumerism, and global mobility require large amounts of energy, mainly covered by fossil fuels that cause significant environmental problems [1,2]. The promotion of hydrogen as a clean energy vector could provide a very efficient strategy for realizing real decarbonization of the current energy system without compromising the continuous growth of the economy [3,4]. Moreover, hydrogen can be obtained from a wide variety of feedstock [5], including hydrocarbon molecules not only from fossil fuels but also from biomass [6] or waste materials [7], as well as ammonia [8] or water [9], among others. In this manner, the use of current mature technologies at the first stages may facilitate a progressive transition to real green hydrogen; meanwhile, other complementary technologies for its transport and storage can be developed to an acceptable standard [10,11]. During this period, which probably will take some decades, pure hydrogen will not be directly produced but will be mixed with other gases such as carbon monoxide, carbon dioxide, nitrogen, or steam. Therefore, additional separation steps will be required to adjust its purity for each application. Among diverse technologies, the use of H_2_-selective membranes provides substantial advantages against other options, such as simplicity, low energy requirements, flexible operation, and moderate cost [12,13]. Moreover, process intensification could be reached by combining both chemical reaction and separation in a unique device, a membrane reactor, where the membrane continuously extracts almost-pure hydrogen from the reactive area [14,15,16].

In this context, fully dense Pd membranes are especially suitable for these applications due to their ideal complete selectivity toward hydrogen, relatively high permeability, and the possibility to work at high temperatures as usually required for most of the reactions [17,18]. A key aspect for these membranes is to reduce as much as possible the thickness of the Pd film to save costs and increase the permeate flux [19,20]. One of the most frequent strategies is to consider a composite structure for the membrane, in which the palladium is deposited as a thin layer onto a porous substrate that provides the required mechanical resistance for the set [12,21]. However, more staked layers can also be generated to modify the original properties of the porous support [22,23] or protect the H_2_-selective film against certain molecules or particular operating conditions [24,25]. In those cases, the final mechanical properties of the system should be carefully addressed under operation because the possibility of a dramatic failure of the membrane noticeably increases with the number of stacked layers made of different materials.

Moreover, an eventual unacceptable cost for membranes with a certain number of layers or materials must also be considered for their industrial implementation in multiple processes. In this context, an extremely facile, low-cost, but effective technique for modifying porous alumina substrates with a graphite lead pencil was proposed for the first time by Hu et al. [26]. They achieved membranes with Pd thickness of around 5 μm by conventional electroless plating (ELP), permeate of 25 m^3^ m^−2^ h^−1^ (at 1 bar and 450 °C), and ideal H_2_/N_2_ separation factor of up to 3700. The same authors followed this strategy for obtaining bi-functional membranes in which the alumina support is doped with Ni particles to prevent the presence of residual amounts of carbon monoxide in the permeate stream [27]. Terra et al. [28] used a similar strategy for the generation of a graphite-based intermediate layer but manually spreading the powder obtained by the milling of a commercial 2B pencil onto the outer surface of alumina hollow fibers. This interlayer allows 25% thinner Pd films to be made with complete H_2_–N_2_ selectivity and a permeance of 1 × 10^−6^ mol m^−2^ s^−1^ Pa^−1^ (at 450 °C). Wei et al. also applied a pencil coating to modify the original surface of macroporous stainless steel supports [29]. They detected some delamination problems but finally reached a Pd thickness of around 7 μm and H_2_ permeance of 4.43 m^3^ m^−2^ h^−1^ kPa^−0.5^ (at 450 °C) after applying vacuum during the intermediate layer incorporation. However, these membranes also evidenced limited ideal H_2_/N_2_ separation factors below 150. It should be noted that all these membranes containing graphite as interlayer were always tested by applying the higher pressure on the outer surface of the membrane, where the Pd film is placed. Thus, all membranes work under the most favorable conditions, with only compression stress that eventually protects any delamination.

Besides the intrinsic material compatibility of stacked layers in any composite membrane, the technology applied for the deposition of the top Pd film could also noticeably improve the mechanical resistance of the overall system. In the last years, a modification of the traditional electroless plating alternative in which the main reactants are separated by the wall of the porous support during the entire deposition process has exhibited very promising results in terms of Pd-thickness reduction and mechanical stability [17,22,30]. This alternative, denoted as electroless pore-plating (ELP-PP), has been satisfactorily applied onto both raw ceramic and metallic porous substrates or modified ones with different intermediate layers such as mixed iron–chromium oxides [31], ceria [22], or SBA-15 [32].

The present study tries to combine, for the first time, an adequate porous stainless steel (PSS) substrate modification with graphite lead as an interlayer with the typical mechanical strength of the Pd film provided by ELP-PP. In contrast to other materials used as intermediate layer when preparing previous ELP-PP membranes, graphite has a greater ductility, thermo-electrical conductivity, and extreme resistance against thermal shocks while presenting a relatively low-cost. Moreover, previous studies in which this intermediate layer based on graphite was deposited onto tubular PSS supports evidenced some limitations for reaching ultra-high H_2_ selectivities, as previously mentioned. Therefore, we consider that this contribution could provide further insights and possibilities for this particular type of H_2_-selective composite membrane. This manuscript addresses the membrane fabrication and also evaluates the membrane’s permeation behavior under a wide variety of operating conditions, including contrary permeation flux directions. This point is especially interesting, since few studies have carried out permeation experiments at unfavorable conditions in terms of tensile stress generation, for example collecting the permeate flux from the outer side of tubular membranes where both intermediate layer and Pd film are placed on the external surface of the porous support.

## 2. Materials and Methods 

### 2.1. Membrane Preparation

In this work, tubular porous stainless steel (PSS) tubes with 0.1 μm media grade and 1⁄2” outside diameter, purchased from Mott Metallurgical Corp. (Farmington, CT, United States of America), were used as support for the Pd composite membranes’ preparation. The original tube of around 60 cm in length was cut into shorter pieces of 30 mm. After that, the general procedure followed for the preparation of the membranes consisted of five successive steps: (i) preliminary cleaning, (ii) support calcination (12 h at 600 °C), (iii) incorporation of graphite as an intermediate layer, (iv) seeding with homogeneously distributed Pd nuclei, and (v) deposition of a palladium film by electroless pore-plating (ELP-PP). Details about the first two general steps, in which the supports were cleaned and calcined, can be found in previous works [17,31].

The intermediate layer was generated by using a graphite lead taken from a 2B pencil according to the original procedure described by Hu et al. [26]. Specifically, the external surface of the PSS substrates was first directly painted with a commercial 2B pencil up to achieve a homogeneous dark gray color but with a significant amount of graphite that was weakly sticky. Then, the excess material was removed by dry brushing with a clean cloth, only leaving the graphite particles just placed inside the deepest pores of the raw support. Cleaning was complete when the textile did not get significantly dirty after this process. All supports modified in this way were then calcined at 500 °C for 5 h to ensure the complete removal of binders and waxes, thus guaranteeing better stability for later stages.

Finally, substrate activation with both Pd nuclei and Pd deposition was carried out based on the well-established electroless pore-plating alternative [12,17,33]. Accordingly, the solutions containing the Pd source and the reducing agent were fed from opposite sides of the support with the aim that they will be present and reactive just in the pores or surrounding areas [30,34,35].

(1) $$Pd^{2+}+N_{2}H_{4}\;\overset{NH_{4}OH}{\to }Pd^{0}+N_{2}$$

Palladium chloride was always used as the metal source but highly diluted (0.1 g/L) into an acidic aqueous solution (HCl, 0.1 mL/L) for the activation step or moderately diluted (5.4 g/L) into a basic one containing ammonium hydroxide (390 mL/L) and ethylenediaminetetraacetic acid (70 g/L) for the deposition itself. On the contrary, a mixture of hydrazine (0.2 M) and ammonia (2.0 M) always formed the reducing solution. These solutions were used at room temperature or 50 °C for each case, i.e., activation or deposition step. However, it should be noted that activation is carried out only once for 2 h, but several recurrences for the Pd deposition are usually required. These ELP-PP cycles were performed until the membrane weight gain became negligible, thus suggesting that a fully dense membrane was reached, and the different solutions could not reach each other because of the blockages of the pores with palladium. This fact was confirmed by gas tightness with helium when the membrane was immersed into ethanol and maintained at room temperature under 3 bar for at least 30 min.

### 2.2. Characterization

The morphology of all samples included here was characterized entirely with a scanning electron microscope Philips XL30 ESEM (Eindhoven, The Netherlands). The external membrane surface was observed before and after incorporating graphite and palladium layers to analyze the uniformity and eventual presence of defects. Furthermore, gravimetric analyses were also used to determine the deposited amount of each material in the membrane, graphite and palladium, by an electronic balance Kern and Sohn ABS-4 (Balingen, Germany) with a precision of 1.0 × 10^−4^ g. Thus, their average layer thicknesses were estimated from these data by assuming a homogeneous distribution on the external surface of the supports. The obtained values were compared with the real thickness obtained from the analysis of cross-sectional SEM images.

**Figure 1:**
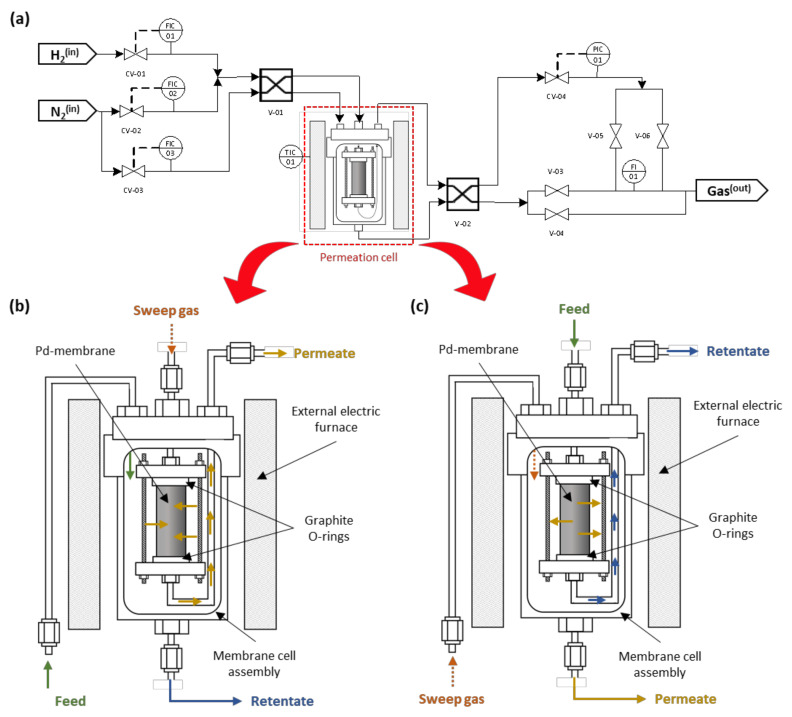
Basic scheme of the permeation setup (**a**) and possible configurations for permeation experiments: mode out–in (**b**) and mode in–out (**c**).

Besides the morphology of the membranes, their permeation capacities with pure gases (H_2_, N_2_) and their mixtures (N_2_ content in the range 5%–40%) were also measured using a homemade facility, depicted in Figure 1 and used in previous studies [22,35].

In essence, the membrane was placed inside a 316L SS vessel rounded by an electrical furnace to operate in the range 350–450 °C. Two graphite O-rings were used to ensure good sealing of the membrane, maintaining both permeate and retentate streams completely separated during the complete set of experiments. The feed stream could be properly adjusted by several Bronkhorst Hi-Tech mass-flow controllers (Ruurlo, The Netherlands) of maximum capacity 400 NmL min^−1^ mounted in independent H_2_ and N_2_ inlet lines. These lines were joined in a unique inlet tube to feed the permeate cell. At this point, it should be noted that two different four-way valves (V-01 and V-02) were mounted in the system to direct the feed stream at the lumen or shell side of the membrane, as later will be explained in detail. On the other hand, permeate or retentate flow rates were measured using an additional Hi-Tech mass-flow meter of maximum capacity 200 NmL min^−1^. In order to avoid any uncertainty while measuring very low fluxes for any retentate of the permeate streams, this equipment was replaced in those cases by a Ritter Mili-GasCounter able to detect volumetric flow-rates from 1.67 × 10^−2^ mL min^−1^. Finally, the equipment counted on the help of a gas chromatograph (GC), Varian CP-4900, with a thermal conductivity detector (TCD) and two analytical columns (Molsieve 5 A° and PoraPLOT-Q) to analyze the composition of both permeate and retentate streams when testing gas mixtures. For the entire set of experiments carried out at pressure driving forces in the range 0.5–2.0 bar (controlled by a Bronkhorst High-Tech EL-PRESS back-pressure regulator – Ruurlo, The Netherlands-), the permeate stream was always maintained at atmospheric conditions, and no sweep gas was used. At this point, it should be noted that two different operation modes were studied in which the function of the position of the aforementioned four-way valves indicated:

i) Mode out–in: The gas is fed to the shell side, thus meeting first the Pd film that extracts part of the gas into the lumen side of the membrane. In this configuration, the adherence of diverse layers is relatively ensured due to the compression stress caused by the higher feed pressure.
ii) Mode in–out: In this case, the feed is introduced to the inner membrane side, thus first passing through the porous support before meeting the Pd film and permeating to the shell side. At these conditions, an inevitable tensile stress is produced between support and the Pd layer, affecting the overall mechanical resistance and possibly causing a dramatic deterioration of the composite membrane by delamination.

## 3. Results and Discussion

### 3.1. Membrane Morphology

**Figure 2:**
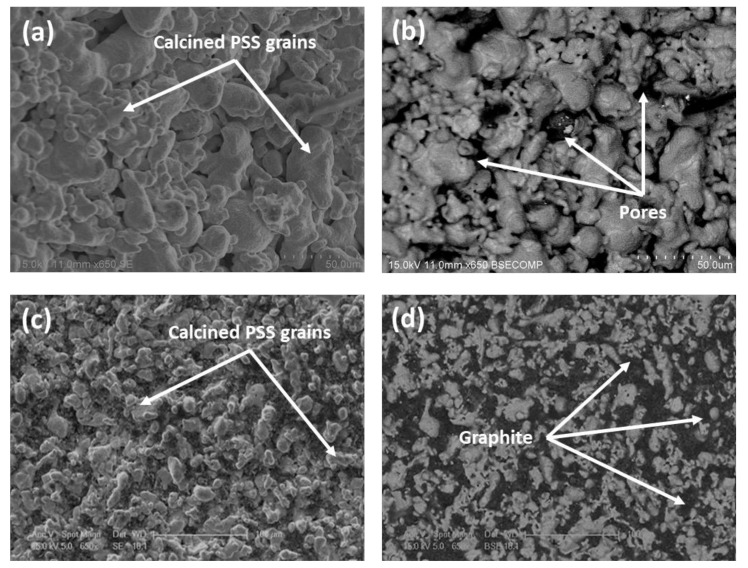
Top-view SEM images of commercial porous stainless steel (PSS) support after modification by calcination in air at 600 °C for 12 h (**a**,**b**) and incorporation of graphite lead particles (**c**,**d**).

The morphology of the composite membranes prepared in this work was analyzed just after incorporating each stacked layer onto the raw PSS support. Figure 2 shows the top-view images of the stainless-steel support, before and after the graphite incorporation. These images were taken by using both secondary electron (Figure 2a,c) and backscattered electron detectors (Figure 2b,d) to analyze more clearly not only the external morphology of the samples but also their composition and material distribution along the surface, respectively.

As can be seen, the calcined supports (Figure 2a,b) present a relatively high roughness with a wide pore-size distribution, at least in their pore mouths. This morphology is very similar to commercial substrates despite generating oxide around the stainless-steel particles by the thermal treatment of calcination [31]. However, this morphology noticeably changes after incorporating the graphite lead (Figure 2c,d). Carbon particles remain preferentially inside the external pores, thus partially blocking the original biggest superficial pores of the substrate and generating a new porous structure with smaller average pore sizes. The starting hypothesis is that these smaller pores could be more easily closed by palladium than the original bigger ones, promoting a relatively thin film with high H_2_ perm-selectivity. Moreover, the initial high roughness of calcined PSS supports was also partially smoothed after incorporating this new intermediate layer.

**Figure 3:**
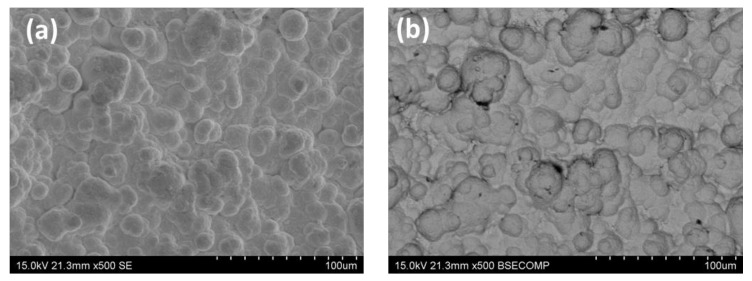
SEM images of the external surface after the palladium incorporation by electroless pore-plating (ELP-PP): (**a**) SE and (**b**) BSE.

Then, the morphology of the final composite membranes reached after the Pd incorporation by ELP-PP was also analyzed by SEM. The characteristic top-view images of this membrane type are collected in Figure 3. Despite the preferential incorporation of the palladium particles just inside the pores of the support or their surrounding areas when using the ELP-PP alternative, in this case, a continuous Pd film that covers the entire surface of the modified supports with graphite can be observed by both SE (secondary electrons) and BSE (back-scattering electrons) images. This morphology, with a spherical grain growth of palladium deposits (Figure 3a), is certainly similar to the ones reached for other ELP-PP membranes in which diverse ceramic intermediate layers were used [32,35]. It can be explained by the presence of a certain pore size distribution in the porous substrates due to the irregularity of the SS grains and the graphite particles. In this manner, the solutions containing the reducing agent (hydrazine) and the metal source (palladium) can probably meet through the smallest pores but not inside the biggest ones. In fact, the higher diffusion velocity of hydrazine toward the metal solution means that the reducing agent reaches the external surface in a relatively short time, where the following autocatalytic reaction initiates:

(2) $$2\mathrm{Pd}{\left({\mathrm{NH}}_{3}\right)}_{4}^{2+}+N_{2}H_{4}+4{\mathrm{OH}}^{-}\to 2{\mathrm{Pd}}^{0}+8{\mathrm{NH}}_{3}+{\;N}_{2}+4H_{2}O$$

Additionally, Figure 3b denotes good compactness of the metal particles and homogeneity along with the entire top film with a palladium content over 95%, as evidenced by EDX analysis. Based on these results evidencing a homogeneous deposition of the palladium around the external surface of modified supports, the palladium thickness can be directly estimated as a uniform outer layer from gravimetric measurements. This value, denoted in the present work as *t_Pd_^e^*, was around 17 μm, which is close to other values obtained when using the ELP-PP procedure to synthesize Pd membranes with intermediate layers such as CeO_2_ [35] or SBA-15 [32].

**Figure 4:**
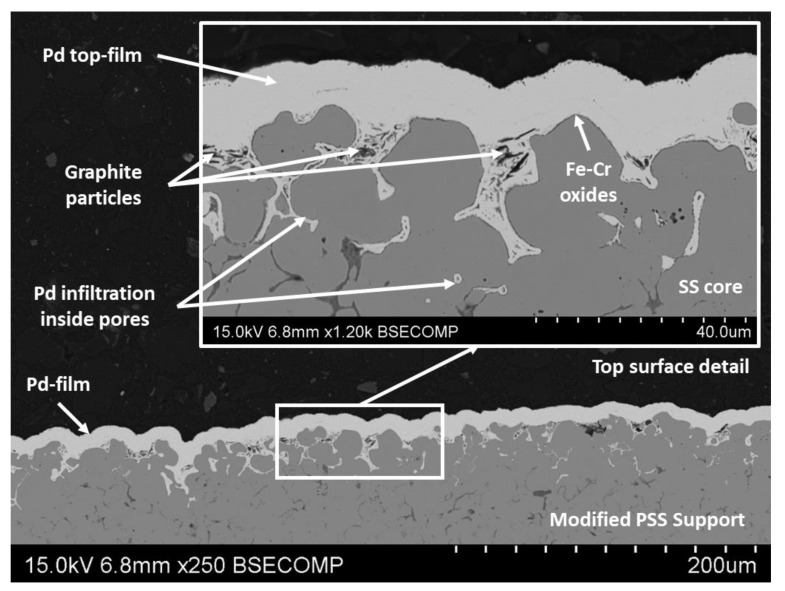
Cross-section of the Pd composite membrane at various magnifications.

More interesting is the analysis of typical cross-sectional views for the new ELP-PP membranes presented in this study with an intermediate graphite layer (Figure 4). The real Pd thickness (*t_Pd_^t^*) can be determined more accurately in these images and the particular distribution of the stacked layers can be analyzed. First, the good continuity of the external Pd film is evident as previously found when analyzing the SEM top-views. However, specific insights can be extracted from the analysis of the image taken at higher magnification. Each material that forms the final composite membrane can be distinguished. The thicker layer, placed on the bottom area of the SEM image, corresponds to the bulk stainless steel support; it indicates the porous stainless steel. Close to the external surface of this support, a very thin dark gray layer with an average thickness below 0.5 μm can also be observed. This layer is made of Fe–Cr oxides generated during the air calcination step at 600 °C for 12 h. It should be noted that these oxides are present not only on the external surface of the support but also inside most of the pores between SS grains. However, due to the low thickness of this first intermediate layer, only the average size of the smallest pores was noticeably reduced. The biggest ones could be modified after incorporating graphite, which can be observed in the figures as dark gray non-spherical particles with a large size distribution. These particles were preferentially placed just inside the mouth of the biggest pores of the calcined support, thus reducing their average size.

A deeper discussion is needed for the top Pd film observed as a light gray layer on the cross-sectional views. This layer presents a true external thickness in the range of around 8–12 μm if it is measured from the crests of the modified support. However, palladium also penetrates some of the pores up to around 35 μm in depth. This is caused by the intrinsic characteristics of the ELP-PP process used for its incorporation, where the chemical reaction between palladium ions and hydrazine (Equation (2)) starts just inside most of the pores, especially in the case of their size becoming limited. This particular distribution of the palladium justifies the differences previously estimated by gravimetric analysis and SEM.

Moreover, it should be noted that the above-mentioned Pd infiltration into most of the external pores of the support could help to improve the adherence of this layer. Consequently, the mechanical resistance of the composite membrane against unfavorable operating conditions, i.e., permeation tests from the inner to the outer side of the membrane in which tensile stress is generated, can also be improved. This potential benefit will be furtherly discussed later while analyzing the permeation behavior of these membranes.

### 3.2. Permeation Behavior

As mentioned before, while describing the experimental procedure to prepare the membranes, preliminary gas bubble tests with helium at room temperature were carried out to ensure enough gas tightness up to 3 bar and to validate the quality of the palladium film. However, it is widely known that additional experiments at different operating conditions closer to the expected ones in real industrial independent separators or membrane reactors are also required. In this context, the present section analyzes in detail the results obtained after performing various permeation tests with pure gases and binary mixtures for a wide variety of experimental conditions, including pressure, temperature, and permeate flux direction (operating mode).

#### 3.2.1. Pure Gases: N_2_ and H_2_

**Figure 5:**
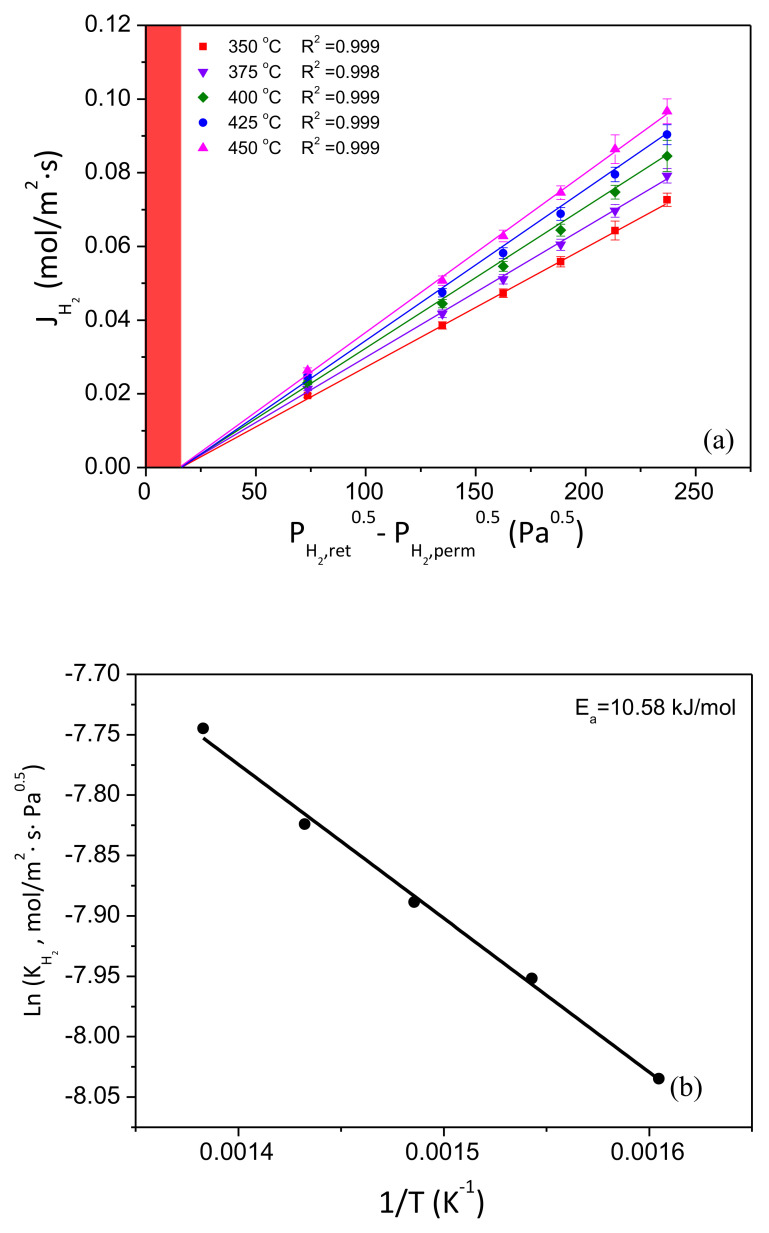
Permeation behavior for out–in operating mode: (**a**) Overall pressure and temperature effects on H_2_ permeate fluxes (deviation from origin interception marked as a red vertical band) and (**b**) determination of the activation energy.

First of all, pressure and temperature effects on the permeation capacity of these membranes were evaluated throughout a series of permeation experiments with pure gases, N_2_ and H_2_, according to the out–in operation mode. These results have been collected in Figure 5. The H_2_ permeate flux is represented against the pressure driving force $\left(P_{H2,ret}^{0.5}-P_{H2,perm}^{0.5}\right)$ for temperatures in the range 350–450 °C (Figure 5a) and calculated H_2_ permeances are correlated with the inverse of the temperature to determine the activation energy of the membrane (Figure 5b). It should be noted that, in all cases, a complete gas tightness against nitrogen was observed at these conditions, thus suggesting an almost ideal complete selectivity to hydrogen according to the available detection limit of the experimental setup (1.67 × 10^−2^ mL min^−1^).

Moreover, it is also important to address that both reproducibility and thermal stability of membranes were previously ensured by several permeation tests before considering further experiments. Particularly, three different membranes were prepared following an analogous experimental procedure, and they were tested under diverse thermal cycles. Each membrane was first heated up to 400 °C under inert atmosphere, measuring the permeate flux after feeding independent single gases, N_2_ and H_2_, at pressures ranged from 0.5 to 2.5 bar before cooling down again up to room temperature under inert conditions. This process was repeated at least five times to ensure adequate membrane resistance and the reliability of permeate fluxes. Further details about the results reached in these experiments can be found in Supplementary Figure S1 as supplementary information. In general, only slight deviations in permeation data were obtained for the entire set of experiments and were assumed as reasonably acceptable. Deviations below 5% were found between each data point taken at similar experimental conditions. In this manner, it is possible to state an adequate reproducibility of permeate values and enough mechanical resistance of the composite membranes against the experimental conditions. Consequently, the membrane GRAPH#01 was selected as reference to continue the analysis of its permeation behavior. In this context, while analyzing in detail the general relationship between H_2_ fluxes and pressure driving forces (shown in Figure 5a), a clear linear trend was observed for the entire range of temperatures. This fact, together with the absence of nitrogen detected in the permeate side during previous experiments carried out with this gas at analogous conditions, confirms the absence of defects in the palladium layer under those conditions. Thus, H_2_ diffusion through the bulk Pd can be considered as the rate-determining step, as described by Sieverts’ law and typically occurs in most Pd composite membranes [19]. At these conditions, the diffusion rate is proportional to the concentration of hydrogen atoms on opposite sides of the metal surface and the hydrogen concentration is proportional to the square root of the hydrogen pressure. On the contrary, the presence of defects and pinholes usually favors other permeation mechanisms different from solution diffusion (i.e., Knudsen diffusion or Poiseuille mechanism) and the pressure exponent should be replaced by other values ranged from 0.5 to 1.0 in order to maintain a good linear fitting. Particularly, n-values of greater than 0.5 when working with Pd films thicker than 5 μm can be attributed to a noticeable permeation of hydrogen through small pore-like defects or pinholes without the necessity of being dissociated, dissolved, and diffused through the bulk metal.

However, it is also relevant to mention that the linear trends do not intercept the origin, as usual in multiple Pd composite membranes prepared by electroless plating. This particular behavior is characteristic of most ELP-PP membranes reported up to now, and it has been widely discussed in our previous manuscripts [17,30,36]. This effect can be explained by the partial infiltration of palladium into the pores of the support. This particular morphology of the Pd film causes both external and internal surfaces of the layer become noticeably different. The outer surface (as previously shown in Figure 3) is certainly smooth and maintains good contact with the gas phase, thus making the pressure values in the bulk gas phase and the surface of the membrane almost identical. However, the internal one is much more tortuous, and the assumptions of negligible differences between both sides of the Pd film and accuracy of the pressure value for that side are not clear. At these conditions, the calculated pressure driving force from retentate and permeate bulk gas phases can be imprecise, and apparent additional resistances to the permeation process appear. In previous studies, it was demonstrated that this deviation is strongly affected by Pd infiltration into the porous substrate [17,36].

On the other hand, the temperature effect on the H_2_ permeate fluxes is also evident for all performed experiments. Independently of the considered temperature for the range 350–450 °C, the above-mentioned linear trend without a clear intercept within the origin is maintained, but reaching higher fluxes as the temperature increases (Figure 5a). In this manner, H_2_ permeance values increase from 3.24 × 10^−4^ to 4.33 × 10^−4^ mol m^−2^ s^−1^ Pa^−0.5^ when working at the lowest or the highest temperature, 350 or 450 °C, respectively. Figure 5b shows the relationship between all these H_2_ permeance values and the temperature at which they were calculated. As can be seen, really good linearity is obtained as predicted by a typical Arrhenius-type dependence. At these conditions, activation energy around 10.6 kJ/mol was obtained within the standard range of other composite Pd-based membranes reported in the literature [17,37,38].

#### 3.2.2. N_2_–H_2_ Binary Mixtures

**Figure 6:**
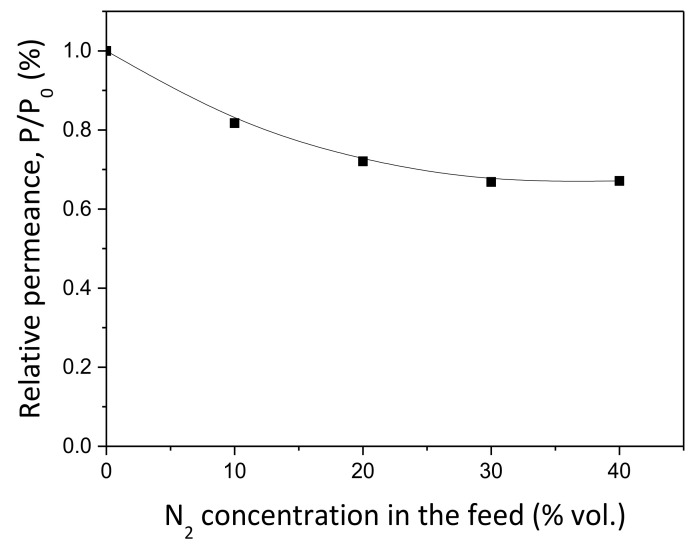
Effect of feed stream dilution with binary H_2_–N_2_ mixtures (out–in operating mode, 400 °C).

After analyzing the permeation behavior when feeding pure H_2_, additional tests with binary H_2_–N_2_ mixtures were also performed to investigate more realistic permeation conditions in which deviation from the theoretical behavior (i.e., concentration–polarization effects) could appear. All these experiments were carried out at a constant temperature of 400 °C while maintaining the out–in configuration for extracting high-purity H_2_ in the permeate side. In this context, Figure 6 collects the experimental results obtained when feeding mixtures in which H_2_ is diluted with different N_2_ concentrations from 0 to 40 vol%. These results are depicted as the relative variation between the permeance values reached for each particular condition and the reference one, 3.75 × 10^−4^ mol m^−2^ s^−1^ Pa^−0.5^, considered when feeding pure H_2_.

First, it should be noted that membrane integrity was maintained during all these experiments since no N_2_ was detected by gas chromatography on the permeate side. However, a clear effect on the H_2_ permeance values appears when increasing the feed dilution with nitrogen. In fact, H_2_ permeances progressively decreased up to around 33% at worst conditions, as the N_2_ content in the feed stream increased from 0 to 40 vol%. This fact cannot be explained by the inherent dilution of the mixture and associated reduction of H_2_ partial pressure. This reduction provokes a consequent decrease of the real pressure driving force, but it was formally considered for data calculation. Then, a certain concentration–polarization effect should be occurring despite the strongly inert character of nitrogen but not at a constant rate for all the analyzed dilutions. First, a significant H_2_ permeance drop of around 28% was obtained when the N_2_ content increases from 0 to 20 vol% in the feed stream. However, dilution up to 30 vol% in N_2_ only generates an additional 5% H_2_ permeance drop concerning the previously given one. This situation appeared to be stabilized for different conditions in which further dilutions were considered.

#### 3.2.3. Effect of the Permeate Flux Direction

The effect of the permeate flux direction in Pd composite membranes prepared by ELP-PP onto modified tubular PSS supports with an intermediate graphite barrier was also analyzed in this work. In all previous tests, the gas was fed to the shell side of the permeation cell, while collecting the permeate from the inner side. This operation mode (out–in) generates compressive stress on the palladium layer and protects the system against possible delamination. Usually, most of the Pd-based membranes from literature are characterized following similar tests, in which permeate flux goes from the outer to the inner side of the membrane [39,40]. However, it could be highly interesting to analyze the results reached in other configurations in which H_2_ permeates in the contrary direction, from the inner to the outer side of the composite membrane. This operating mode, denoted as in–out in the present study, generates certain tensile stress on the top palladium film due to the pressure difference between both system sides. Therefore, these operating conditions could be used as an indirect measurement of the tensile strength of the membrane and, as a consequence, its mechanical resistance. Moreover, it could also be interesting to demonstrate an adequate resistance of the membranes against these operating conditions for their use in membrane reactors, where it would be convenient to place a solid catalyst in the membrane lumen for certain applications in which direct contact between palladium and diverse chemicals should be avoided.

**Figure 7:**
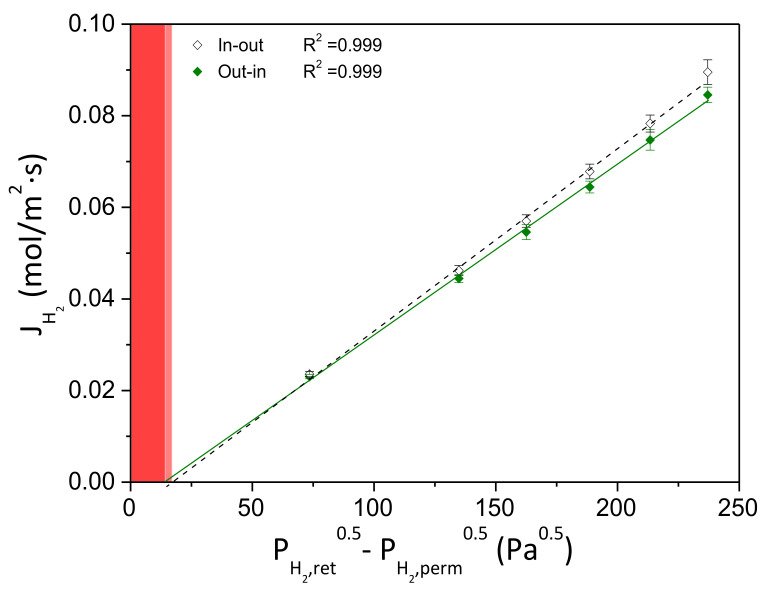
Permeation behavior working at diverse operating modes: out–in (colored symbols) and in–out (hollow symbols). Deviation from origin interception marked as a red vertical band for each case.

In this context, Figure 7 collects the H_2_ permeate fluxes reached at 400 °C in case of working under both operating modes, out–in and in–out.

First, it should be mentioned that no nitrogen was detected for the entire range of pressures despite the new configuration. Thus, an adequate mechanical resistance of the Pd layer and, consequently, of the whole membrane, can be deduced. Moreover, as can be seen, very similar behavior can be observed independently of the followed operating mode. A clear linear relationship between the H_2_ permeate flux and pressure driving forces appears, although with a certain deviation from the origin as previously addressed. This deviation slightly increases in the case of permeating from the inner to the outer side of the membrane (mode in–out). The presence of the porous support on the retentate side, where the highest pressure is applied, can explain this fact. In this manner, the permeate has to pass through the intrinsic porosity of the modified graphite–PSS support and its tortuosity. Moreover, it is possible to find a certain amount of palladium inside the pores, before reaching the fully dense Pd film that is placed on the contrary side. It provokes a certain resistance to the mass transfer and, consequently, a deviation between the measured feed/retentate pressure and the exact value just close to the palladium layer. This particular behavior was widely explained in some previous studies for other ELP-PP membranes [36,41].

As a consequence of the above-mentioned behavior, H_2_ permeance slightly increases for this operating mode (in–out) up to 3.75 × 10^−4^ mol m^−2^ s^−1^ Pa^−0.5^ from the value 4.01 × 10^−4^ mol m^−2^ s^−1^ Pa^−0.5^ reached with contrary permeate flux direction (out–in).

#### 3.2.4. Literature Setting for Permeation Behavior

Finally, Table 1 includes a literature survey about some representative achievements reached for other composite Pd-based membranes to be compared with the results presented in this study. The comparison consists of membranes fabricated by electroless plating or related techniques onto supports made of different materials or various geometries. The wide variety of information about membrane composition, structure, morphology, and permeation experimental details, among others, clearly increases the complexity of a rigorous comparison.

In general, the thinnest selective films have the highest hydrogen permeation, although it is also common to find a limited H_2_ permeation in these cases. In this work, an ideal H_2_/N_2_ separation factor higher than 10,000 was obtained for the entire set of experiments, independently of the operating mode, out–in or in–out. This fact remarks on the excellent mechanical resistance of the ELP-PP membranes with an intermediate layer made of graphite presented in this work. Similar demonstrations for other Pd membranes reported in the literature are certainly scarce. However, the membranes shown here maintained their integrity against diverse permeation flux directions for the entire set of experiments carried out under compressive or tensile strengths generated by the pressure difference between both retentate and permeate sides, thus opening new possibilities for use in a wide variety of reactor configurations. At these conditions, the permeate fluxes reached at ΔP = 1 bar were maintained in the range 3.7–4.8 m^3^ m^−2^ h^−1^.

These values are within the typical ones reached for other completely H_2_-selective membranes prepared by ELP-PP but containing various intermediate layers. However, they seem to be slightly lower than those of other membranes prepared by conventional ELP, mostly onto alumina supports. The initial high surface quality of these supports, in terms of roughness and average pore sizes, makes the incorporation of a thin Pd film easier and, in general, they present a lower Pd thickness and higher permeate fluxes. On the contrary, it should be noted that H_2_ selectivity is usually compromised.

## 4. Conclusions

Previously calcined porous stainless-steel supports with an initial media grade of 0.1 μm were satisfactorily modified by incorporating graphite particles from a lead pencil as an intermediate layer to improve the following incorporation of palladium by electroless pore-plating. The carbon particles remained preferentially inside the external pores of the supports, thus partially blocking the original biggest superficial pore mouths and generating a new porous structure with smaller average pore sizes and lower roughness. After this modification, completely dense Pd layers with an estimated gravimetric thickness of around 17 μm were obtained. However, cross-sectional SEM images revealed a slightly thinner true external thickness in the range 8–12 μm with a certain infiltration of the palladium inside the pores up to around 35 μm in depth. This particular incorporation of the palladium is caused by the intrinsic characteristics of the ELP-PP process, where the chemical reaction between palladium ions and hydrazine initiates inside the pores.

These new membranes exhibit H_2_ permeances in the range 3.24 × 10^−4^ to 4.33 × 10^−4^ mol m^−2^ s^−1^ Pa^−0.5^ when permeating from the outer to the inner side (mode out–in) at temperatures ranging from 350 to 450 °C, respectively. Moreover, an ideal H_2_/N_2_ perm-selectivity ≥10,000 was maintained for the entire set of experiments. Going into detail about the permeation behavior of these membranes, a general linear trend between permeate H_2_ fluxes and pressure-driving forces can be found but without a clear intercept in (0,0) as predicted by the Sieverts’ law. The Pd partial infiltration inside the pore structure of the support during the ELP-PP process explains this deviation, as typically occurs for other similar membranes in which both external and internal Pd surfaces become noticeably different. The outer surface is certainly smooth, with the measured pressure values into the bulk gas phase and the surface of the membrane being almost identical. However, the internal one is much more tortuous, and the assumptions of negligible differences between both sides are not clear. At these conditions, the calculated pressure-driving force from retentate and permeate bulk gas phases can be imprecise, and apparent additional resistances to the permeation process appear. The temperature effect on the H_2_ permeate fluxes, according to an Arrhenius-type dependence, was also evident for all performed experiments with activation energy around 10.6 kJ/mol, very close to that of multiple Pd composite membranes.

In the case of testing binary H_2_–N_2_ mixtures, increasing the N_2_ content in the feed stream from 0 to 40 vol%, H_2_ permeances progressively decreased up to around 33% in the worst conditions. A certain contribution of the well-known concentration–polarization effect, despite the strongly inert character of nitrogen, could explain this fact. This effect is not constant for any concentration of the mixture, being more marked for lower dilutions.

Finally, some additional experiments were also performed after reversing the direction of the permeate flux. Certainly, similar results were reached in terms of permeate fluxes and H_2_ selectivity. Therefore, adequate mechanical resistance was practically demonstrated despite operating at the most unfavorable conditions (mode in–out) in which certain tensile stress is generated on the Pd layer.

## Figures and Tables

**Table 1:** Comparison of main characteristics and performance for relevant membranes in literature.

Support	Intermediate Layer	H_2_-Selective Layer			Membrane Performance at ΔP = 1 bar				Ref.
		Incorporation Technique	Composition	*t_Pd_* (μm)	Operating Mode	T (K)	Permeate Flux (m^3^ m^−2^ h^−1^)	α_H2/N2_	
- **^(1)^**	-	Cold-rolling	PdAg	50	in–out	573	1.1 × 10^−2^	-	[42]
PSS ^(1)^	Fe_2_O_3_–Cr_2_O_3_	ELP	Pure Pd	20	out–in	623	3.4	500	[43]
PSS ^(1)^	Fe_2_O_3_–Cr_2_O_3_	ELP-PP	Pure Pd	11–20	in–out	623–723	1.1–2.2	∞	[30]
PSS ^(1)^	Al_2_O_3_	ELP	Pure Pd	5	out–in	673	3.3	500	[44]
PSS ^(1)^	CeO_2_	ELP	Pure Pd	13	out–in	773–823	10.2–22.2	∞	[45]
PSS ^(2)^	CeO_2_	ELP	PdCu	8	-	723	5.9	2369	[46]
PSS ^(1)^	ZrO_2_	ELP	PdAu	14–27	out–in	673–773	3.8–7.8	4000	[47]
Al_2_O_3_^(1)^	-	ELP-duplex	Pure Pd	2.8/2.5	out–in	773	17.7	14,429	[40]
PSS ^(1)^	Fe_2_O_3_–Cr_2_O_3_/CeO_2_	ELP-PP	Pure Pd	15.4	out–in	673	4.6	≥10,000	[22]
PSS ^(1)^	Fe_2_O_3_–Cr_2_O_3_/CeO_2_	ELP-PP	Pure Pd	15.4	in–out	673	4.8	≥10,000	[22]
PSS ^(1)^	Fe_2_O_3_–Cr_2_O_3_/Pd-doped CeO_2_	ELP-PP	Pure Pd	9.1	out–in	673	5.1	≥10,000	[35]
PSS ^(1)^	Fe_2_O_3_–Cr_2_O_3_/Pd-doped CeO_2_	ELP-PP	Pure Pd	9.1	in–out	673	5.3	≥10,000	[35]
Al_2_O_3_^(1)^	Graphite	ELP	Pure Pd	5	out–in	673	22.7	3100	[26]
NiO/Al_2_O_3_^(1)^	Graphite	ELP	Pure Pd	5	out–in	623–723	16–20	500–750	[27]
Al_2_O_3_^(1)^	Graphite	ELP	Pure Pd	5	out–in	623–723	14–18	5500–7500	[27]
Al_2_O_3_^(1)^	Nontronite-15A	ELP	Pure Pd	5	out–in	623–723	11.3–16.9	2000–3700	[48]
Al_2_O_3_^(3)^	Graphite	ELP	Pure Pd	1.81	out–in	573–723	0.8–8.9	∞	[28]
PSS ^(1)^	Graphite	ELP	Pure Pd	7	out–in	623–723	12–18	60–120	[29]
PSS ^(1)^	Fe_2_O_3_–Cr_2_O_3_/Graphite	ELP-PP	Pure Pd	17	out–in	623–723	3.7–4.8	≥10,000	This work
PSS ^(1)^	Fe_2_O_3_–Cr_2_O_3_/Graphite	ELP-PP	Pure Pd	17	in–out	673	4.4	≥10,000	This work

Membrane geometry: ^(1)^ tubular, ^(2)^ disc, and ^(3)^ hollow fiber.

## Supplementary Materials

The following are available online at https://www.mdpi.com/2077-0375/10/12/410/s1, Figure S1: Validation of permeation measurements by testing three different membranes prepared at analogous conditions (PSS/GRAPH/Pd) under consecutive thermal cycles.
