# Supplementary material for: Effective H2 Separation through Electroless Pore-Plated Pd Membranes Containing Graphite Lead Barriers
Source: Membranes (Basel). 2020 Dec 10;10(12):410. doi: 10.3390/membranes10120410 (PMC7764324; doi:10.3390/membranes10120410)
Supplement: Supplementary file 1 [file membranes-10-00410-s001.pdf]

membranes

Effective H_2 Separation through Electroless Pore-Plated Pd Membranes Containing Graphite Lead Barriers

David Martinez-Diaz [1], Raúl Sanz [2], Alicia Carrero [1], José Antonio Calles [1] and David Alique [1,*]

[1] Department of Chemical, Energy and Mechanical Technology, Rey Juan Carlos University, C/ Tulipán s/n, 28933 Móstoles, Spain; david.martinez.diaz@urjc.es (D.M.-D.); alicia.carrero@urjc.es (A.C.); joseantonio.calles@urjc.es (J.A.C.)

[2] Department of Chemical and Environmental Technology, Rey Juan Carlos University, C/ Tulipán s/n, 28933 Móstoles, Spain; raul.sanz@urjc.es

* Correspondence: david.alique@urjc.es (D.A.); Tel.: +34-91-488-7603

SI. Supplementary Information

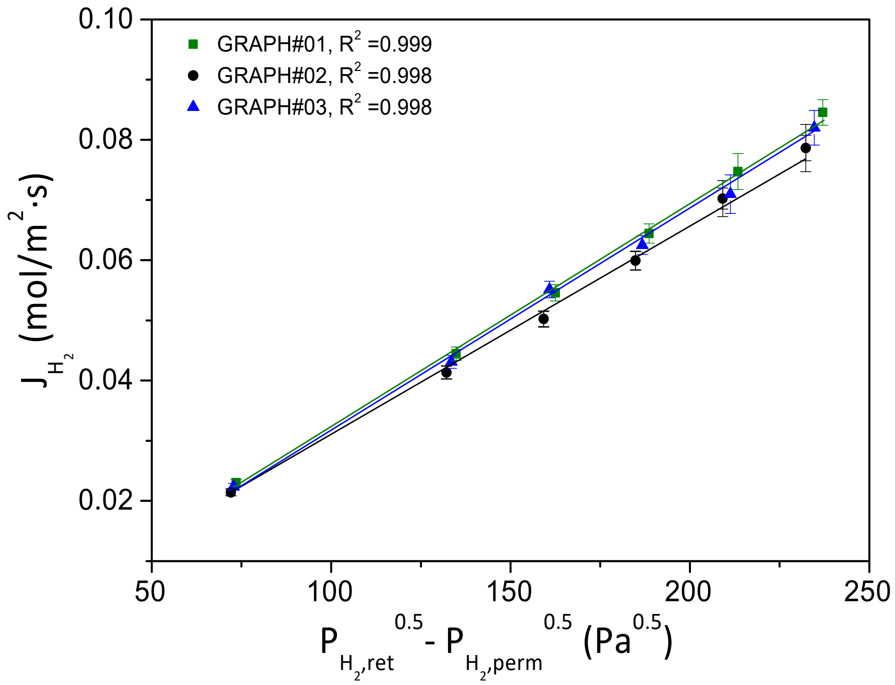

Figure S1. Validation of permeation measurements by testing three different membranes prepared at analogous conditions (PSS/GRAPH/Pd) under consecutive thermal cycle
